# Synergistic effects of metal ion and the pre-senile cataract-causing G98R αA-crystallin: self-aggregation propensities and chaperone activity

**Published:** 2009-10-16

**Authors:** Devendra Singh, Ramakrishna Tangirala, Raman Bakthisaran, Mohan Rao Chintalagiri

**Affiliations:** Centre for Cellular and Molecular Biology, Council of Scientific and Industrial Research, Hyderabad, India

## Abstract

**Purpose:**

αA- and αB-crystallins are abundantly present in the eye lens, belong to the small heat shock protein family, and exhibit molecular chaperone activity. They are also known to interact with metal ions such as Cu^2+^, and their metal-binding modulates the structure and chaperone function. Unlike other point mutations in αA-crystallin that cause congenital cataracts, the G98R mutation causes pre-senile cataract. We have investigated the effect of Cu^2+^ on the structure and function of G98R αA-crystallin.

**Methods:**

Fluorescence spectroscopy and isothermal titration calorimetry were used to study Cu^2+^ binding to αA- and G98R αA-crystallin. Circular dichroism spectroscopy was used to study secondary and tertiary structures, and dynamic light scattering was used to determine the hydrodynamic radii of the proteins. Chaperone activity and self-aggregation of the wild type and the mutant protein in the absence and the presence of the metal ions was monitored using light scattering.

**Results:**

Our fluorescence quenching and isothermal titration calorimetric studies show that like αA-crystallin, G98R αA-crystallin binds Cu^2+^ with picomolar range affinity. Further, both wild type and mutant αA-crystallin inhibit Cu^2+^-induced generation of reactive oxygen species with similar efficiency. However, G98R αA-crystallin undergoes pronounced self-aggregation above a certain concentration of Cu^2+^ (above subunit to Cu^2+^ molar ratio of 1:3 in HEPES-NaOH buffer, pH 7.4). At concentrations of Cu^2+^ below this ratio, G98R αA-crystallin is more susceptible to Cu^2+^-induced tertiary and quaternary structural changes than αA-crystallin. Interestingly, Cu^2+^ binding increases the chaperone-like activity of αA-crystallin toward the aggregation of citrate synthase at 43 °C while it decreases the chaperone-like activity of G98R αA-crystallin. Mixed oligomer formation between the wild type and the mutant subunits modulates the Cu^2+^-induced effect on the self-aggregation propensity. Other heavy metal ions, namely Cd^2+^ and Zn^2+^ but not Ca^2+^, also promote the self-aggregation of G98R αA-crystallin and decrease its chaperone-like activity.

**Conclusions:**

Our study demonstrates that unlike wild type αA-crystallin, G98R αA-crystallin and its mixed oligomers with wild type protein are vulnerable to heavy metal ions. Our study provides insight into aspects of how environmental factors could augment phenotype(s) in certain genetically predisposed conditions.

## Introduction

αA- and αB-crystallins, members of the small heat shock protein family [1], are abundantly present in the eye lens. αB-crystallin is also significantly expressed in non-lenticular tissues such as the heart, muscle, kidney, and brain whereas αA-crystallin is expressed in traces of the spleen and thymus [2]. They form homo- and hetero-oligomers and exhibit molecular chaperone-like activity in preventing the aggregation of other proteins [3-7]. Interestingly, studies from our laboratory as well as those from others show that both αA- and αB-crystallins exhibit pronounced changes in structural and chaperone-functional aspects upon interacting with metal ions such as Cu^2+^ and Zn^2+^ [8-10]. It is also important to note that these metal ions have been reported to accumulate in age-related cataractous lenses [11-15]. Increasing numbers of point mutations in α-crystallins have been reported to be associated with cataract [16-31]. However, the effect of heavy metal ions in general and Cu^2+^ in particular (due to its redox active nature) on the structure and chaperone functional aspects of disease-causing point mutants is not yet addressed.

Most point mutations in α-crystallins are known to cause dominant negative congenital cataract either alone or in association with other pathological conditions such as myopathy [16-31]. Unlike other mutations in α-crystallins that cause congenital cataract, the G98R mutation in αA-crystallin has been reported to manifest in onset of cataract at about 16 years of age [22]. Our earlier studies [32,33] addressed the structural and functional differences between the wild type and mutant protein. Our studies showed that the G98R mutation in αA-crystallin leads to folding defects, resulting in inclusion bodies formation (irreversible aggregation) in the crowded milieu of cells (e.g., in *Escherichia coli*). G98R αA-crystallin does not exhibit chaperone-like activity toward dithiothreitol (DTT)-induced aggregation of insulin, and the mutation leads to destabilization of the protein toward heat- and urea-induced unfolding and increased susceptibility to proteolysis. A study from another laboratory has reported that the chaperone-activity of G98R αA-crystallin is target protein-dependent [34]. Though the G98R mutation results in folding-defective, aggregation-prone αA-crystallin, the mutation-affected individuals develop early onset (pre-senile) cataract and not congenital cataract. We believe that the formation of mixed oligomers [33] or some environmental factors could be responsible for such pre-senile onset of the phenotype.

As mentioned earlier, metal ions such as Cu^2+^, Cd^2+^, Zn^2+^, and Ca^2+^ are known to be present in the eye lens, and their levels increase with age or in cataractous lenses [26-30]. In the present study, we have addressed how such ionic interactions or complex formation (metal ion binding) coupled with the G98R mutation affect the structure and function of αA-crystallin. Such investigations have not been performed earlier. The results of our study should prove useful in understanding how environmental factors in general can influence the manifestation of mutant phenotype(s).

## Methods

### Materials

Insulin, citrate synthase (CS), dithiothreitol (DTT), coumarin-3-carboxylic acid (3-CCA), CdCl_2_, and sodium salts of fluorescein and N-acetyl tryptophanamide (NATA) were obtained from Sigma (St. Louis, MO). The sodium salt of 2, 6 dichlorophenol-indophenol (DCI) was obtained from SRL (Mumbai, India). Analytical reagent grade CuCl_2_ was supplied by Qualigens (Mumbai, India). CaCl_2_ and ZnCl_2_ standard solutions were purchased from Fluka (Fluka, Buchs, Switzerland).

### Expression and purification of human αA- and G98R αA-crystallins

Wild type and G98R αA-crystallins were overexpressed and purified as described elsewhere [7,32]. Protein concentrations were determined using an extinction coefficient (ε_0.1%, 280 nm_) of 0.725, which was calculated by a method described by Pace et al. [35]. Both proteins were passed through a PD10 column to remove EDTA, and the buffer was exchanged with either buffer A (20 mM phosphate, pH 7.4, containing 100 mM NaCl) or buffer B (20 mM HEPES-NaOH, pH 7.4, containing 100 mM NaCl).

### Cu^2+^-binding studies

In all Cu^2+^-binding experiments, we have used Cu^2+^ in the presence of glycine as this approach is known to avoid less-specific or non-specific interactions of Cu^2+^ and reveals its tight-binding to protein [36,37].

### Fluorescence spectroscopy

Fluorescence spectra were recorded from 310 to 400 nm using a Hitachi F4500 Fluorescence Spectrophotometer (Hitachi, Tokyo, Japan) with the excitation wavelength set at 295 nm. αA- and G98R αA-crystallin (5 μM subunits, i.e., 0.1 mg/ml in buffer A) were titrated with increasing concentrations of Cu^2+^ (used from a 1 mM CuCl_2_ stock solution complexed with two mole equivalent of glycine) in the range of 0–50 μM. NATA (5 μM), thyroglobulin (0.1 mg/ml), and α-synuclein (0.1 mg/ml; excitation 275 nm; emission 285–350 nm) were used as controls. Fluorescence quenching was calculated using the formula (F_0_-F)/F_0_, where F_0_ and F are fluorescence intensities at 337 nm (in the case of α-synuclein, 300 nm) in the absence and in the presence of specified concentrations of Cu^2+^. Data were fitted by nonlinear regression with hyperbolic function (Equation 1) using GraphPad Prism 4.0 software (GraphPad Software Inc., La Jolla, CA) for overall one-site binding isotherm.

Equation 1

Y=Bmax*X/(Kd+X)

where B_max_ is the maximum binding (reflected by the maximum extent of quenching), X is the Cu^2+^ concentration, and Y is the fluorescence quenching at a given concentration of ligand as described above. K_d_ is the dissociation constant. K_d_ is the equilibrium constant for the reaction, MX=M + X, and is given by

Equation 2

Kd=[M][X]/[MX]

where [M], [X], and [MX] are the equilibrium concentrations of the macromolecule (in this case, the protein αA-crystallin), ligand (Cu^2+^), and protein-ligand complex, respectively. K_d_ is defined as the ligand concentration for half-maximal binding.

### Isothermal titration calorimetry

Isothermal titration calorimetry (ITC) was performed using a VP-ITC instrument (Microcal Inc., Northampton, MA). Aliquots (2 μl) of 1 mM Cu^2+^ in buffer B were injected into the ITC cell containing either buffer B alone or the buffer containing 0.4 mg/ml (approximately 20 μM subunit) of G98R αA-crystallin were injected at 30 °C into the ITC cell. After subtracting the buffer blank from each experimental titration, the integrated heat of each injection was used for fitting to binding models using Microcal Origin 7.0 software. The isotherm could be best fitted with sequential binding model with five sets of binding sites (n=5). The software follows the iterative curve fitting method using a set of equations described below for the sequential binding model.

For “n” number of sequential binding sites, the binding constants (or association constants) K1, K2,…Kn is defined relative to the progress of saturation, so that

Equation 3

K1=[MX1]/[M][X],....Kn=[MXn]/[M][X]

where M is the molar concentration of the macromolecule (unbound) and [X] is the free ligand concentration.

Equation 4

$$\text{[X]=Xt-Mt}\sum_{\text{i-1}}^{\text{n}}\text{iFn}$$

where M_t_ is the bulk macromolecular concentration and X_t_ is the bulk ligand concentration and F_n_ is the fraction of macromolecule having “n” bound ligand.

Equation 5

$$\text{Fn=K1K2}...{\text{Kn[X]}}^{\text{n}}\text{/P}$$

and Equation 6

$${\text{P=1+K1[X]+K1K2[X]}}^{\text{2}}\text{+}...\text{+K1K2}..{\text{Kn[X]}}^{\text{n}}$$

Once the “n” and the fitting parameters, K1 through Kn, are assigned, Equations 4–6 are solved for [X] and Fn, and the heat content (Q) after the i^th^ injection is determined from Equations 7 and 8, which leads into the Marquardt minimization routine.

Equation 7

$$\text{Q}={\text{M}}_{\text{t}}{\text{ V}}_{0}\;\left(\text{F1}\Delta \text{H1 }+\text{ F2}\left[\Delta \text{H1}+\Delta \text{H2}\right]\;+...+\text{ Fn}\left[\Delta \text{H1 }+\Delta \text{H2 }+..\;\Delta \text{Hn}\right]\right)$$

Equation 8

$$\Delta \text{Q}\left(\text{i}\right)=\text{Q}\left(\text{i}\right)\;+{\text{ dV}}_{\text{i}}/{\text{V}}_{0}\left[\{\text{Q(i)+Q(i-1)}\}/\text{2}\right]–\text{Q}\left(\text{i}-\text{1}\right)$$

where V_0_ is the working volume of the ITC cell and ΔH is enthalpy change.

### Cu^2+^-catalyzed generation of hydroxyl radical (OH˙)

Hydroxyl radical generation upon the addition of Cu^2+^ (1 μM) to buffer A containing ascorbate (300 μM) in the absence or in the presence of indicated concentrations of various proteins was studied by monitoring the increase in fluorescence of 3-CCA (100 μM). The fluorescence intensity was measured at 450 nm upon excitation at 395 nm using a Spectramax Gemini XS microplate spectrofluorimeter (Molecular Devices, Sunnyvale, CA).

In another experiment, the generation of reactive oxygen species (ROS) and copper-catalyzed oxidation of ascorbate to dehydroascorbate in the presence and in the absence of proteins was performed as described in an earlier study [8].

### Metal ion-induced self-aggregation

Self-aggregation of αA-crystallin or G98R αA-crystallin (0.1 mg/ml [approximately 5 μM subunit]) or the mixed oligomer (formed by mixing αA- and G98R αA-crystallin in a ratio of 1:1 [w/w] and incubating at 37 °C for 3.5 h) in buffer B at 37 °C was monitored by light scattering with increasing concentrations of different metal ions. Ten minutes after each addition of the metal ion, light scattering was measured using Hitachi F-4000 Fluorescence Spectrophotometer with excitation and emission wavelengths set at 465 nm.

To study the reversibility of aggregation, G98R αA-crystallin, αA-crystallin, and the mixed oligomer (0.1 mg/ml) was incubated for 30 min at 37 °C with 30, 90, and 90 μM Cu^2+^, respectively. Subsequently, 200 μM EDTA was added, and light scattering was monitored for 20 min at 465 nm.

### Chaperone assay

Aggregation of insulin (0.2 mg/ml in 10 mM phosphate buffer, pH 7.4, containing 100 mM NaCl) was initiated by the addition of 20 mM DTT at 37 °C in the absence or in the presence of 0.1 mg/ml (approximately 5 μM subunit) αA- or G98R αA-crystallin with or without 15 μM Cu^2+^. Aggregation of CS (25 μg/ml) in 40 mM HEPES-NaOH buffer, pH 7.4, at 43 °C was studied with indicated concentrations of different metal ions in the absence or in the presence of 20 μg/ml (approximately 1 μM subunit) αA-, G98R αA-crystallin, or the mixed oligomer. Aggregation was monitored by light scattering at 465 nm using Hitachi F-4000 Fluorescence Spectrophotometer that was equipped with a temperature-regulated cuvette holder and stirrer.

### Circular dichroism

Near- and far-ultraviolet (UV) circular dichroism (CD) spectra of 50-μM protein samples (1.0 mg/ml) in buffer B at room temperature were recorded using a JASCO J-815 Spectropolarimeter (Easton, MD) in the absence and in the presence of 150 μM Cu^2+^. All reported spectra are the cumulative average of four scans, smoothed and expressed as the mean residue mass ellipticity after subtraction of the appropriate buffer blank.

### Dynamic light scattering

The hydrodynamic radii (R_h_) of proteins were determined at 25 °C using dynamic light scattering (DLS) at 90° with a Photocor DLS Instrument (Photocor Instruments Inc., College Park, MD). A laser power of 25 mW with a wavelength of 633 nm was used to make the measurements. Protein samples (25 μM) in the absence or in the presence of 75 μM Cu^2+^ were filtered through a 0.22 μm membrane before the measurements. The data were analyzed using Dynals v2.0 software (Tirat, Carmel, Israel).

### Thermal stability

The thermal aggregation of 0.2 mg/ml (approximately 10 μM subunit) of αA- and G98R αA-crystallin in buffer B in the presence or the absence of 30 μM Cu^2+^ was studied by measuring light scattering at 465 nm on a Flurolog-3 fluorescence spectrophotometer (Jobin Yvon, Edison, NJ).

## Results

### Cu^2+^-binding to αA- and G98R αA-crystallins

**Figure 1:**
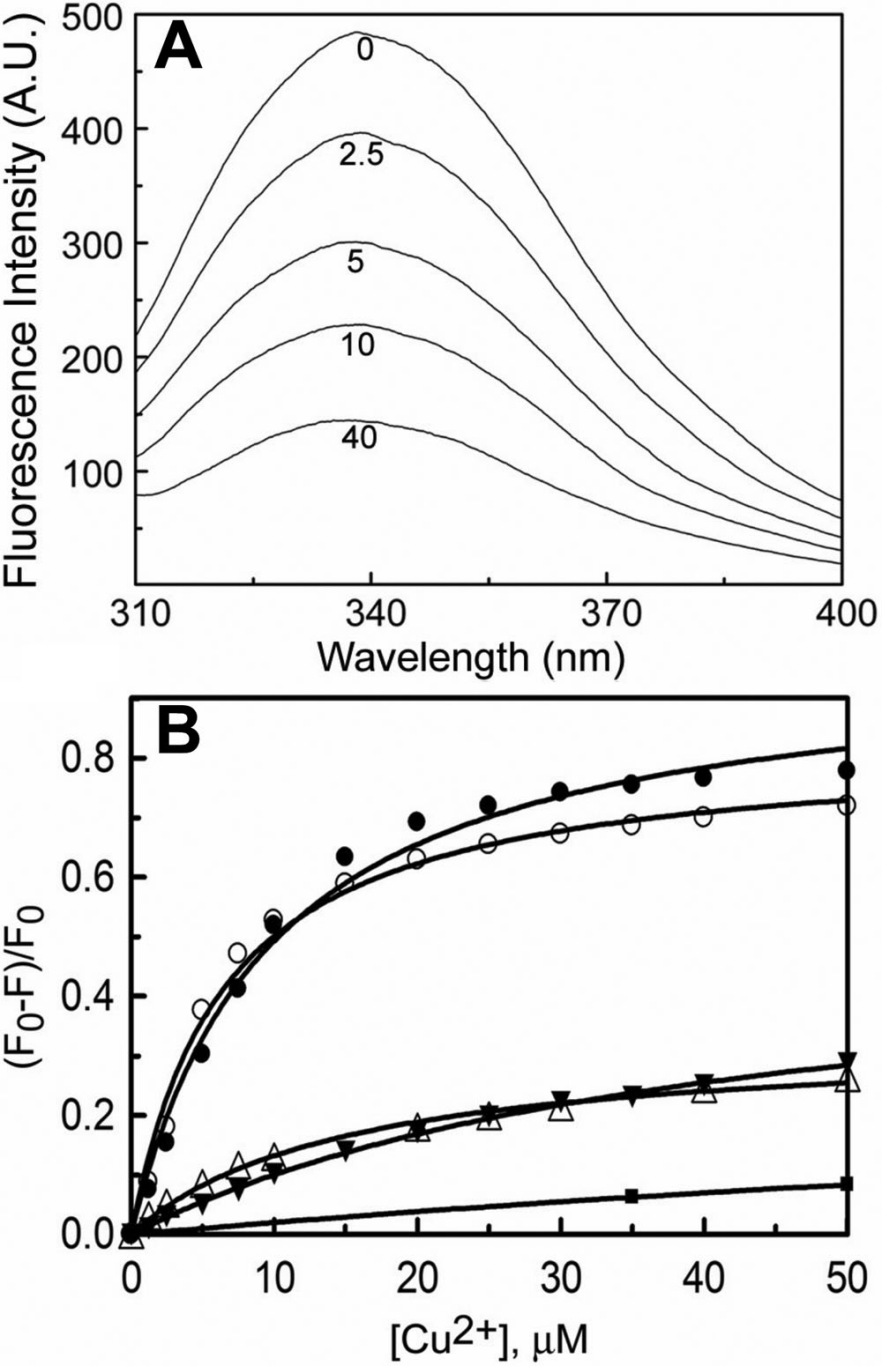
Quenching of intrinsic fluorescence upon binding of Cu^2+^.  **A**: Intrinsic tryptophan fluorescence spectra of 0.1 mg/ml sample of αA-crystallin in buffer A at indicated concentrations (in µM) of Cu^2+^ are shown. **B**: The extent of fluorescence quenching [(F_0_-F)/F_0_] of 0.1 mg/ml αA- (○) and G98RαA-crystallin (●) at 25 °C is shown as a function of Cu^2+^ concentration.  The extent of fluorescence quenching of the controls, 5 μM NATA (■), 0.1 mg/ml of thyroglobulin (△) and α-synuclein (▼) as a function of Cu^2+^ concentration are also shown.  F_0_ and F are the fluorescence intensities at 337 nm in the absence and in the presence Cu^2+^. In the case of α-synuclein which lacks tryptophan residue, fluorescence intensity of tyrosine residues was measured at 300 nm. Both αA- and G98RαA-crystallin exhibit similar extent of fluorescence quenching indicating that they have similar Cu^2+^-binding properties.

We have investigated the binding of Cu^2+^ to the mutant G98R αA-crystallin by fluorescence quenching as well as isothermal titration calorimetry (ITC) as described in our earlier study on Cu^2+^-binding to αA-crystallin [8]. Figure 1A shows increased quenching of the tryptophan fluorescence of αA-crystallin as a function of Cu^2+^ concentration. Similarly, the addition of Cu^2+^ to the sample of G98R αA-crystallin (but not the controls, thyroglobulin [640 kDa], α-synuclein, or NATA) leads to significant fluorescence quenching (Figure 1B). A comparison of the extent of Cu^2+^-induced fluorescence quenching of G98R αA-crystallin with that of αA-crystallin and of the derived dissociation constants shows that both the proteins exhibit similar Cu^2+^-binding properties (Figure 1B and Table 1).

**Table 1:** Comparison of binding constants of Cu^2+^-αA-crystallin interactions determined by fluorescence spectroscopy and isothermal titration calorimetry.

**Protein**	**K_d(app)_**		**K_d(real)_**	
	**Fluorescence**	**ITC**	**Fluorescence**	**ITC**
αA-crystallin	6.4×10^−6^	12.0×10^−6^	16.6×10^−12^	31.2×10^−12^
G98R αA-crystallin	9.8×10^−6^	4.7×10^−6^	25.5×10^−12^	12.2×10^−12^

**Figure 2:**
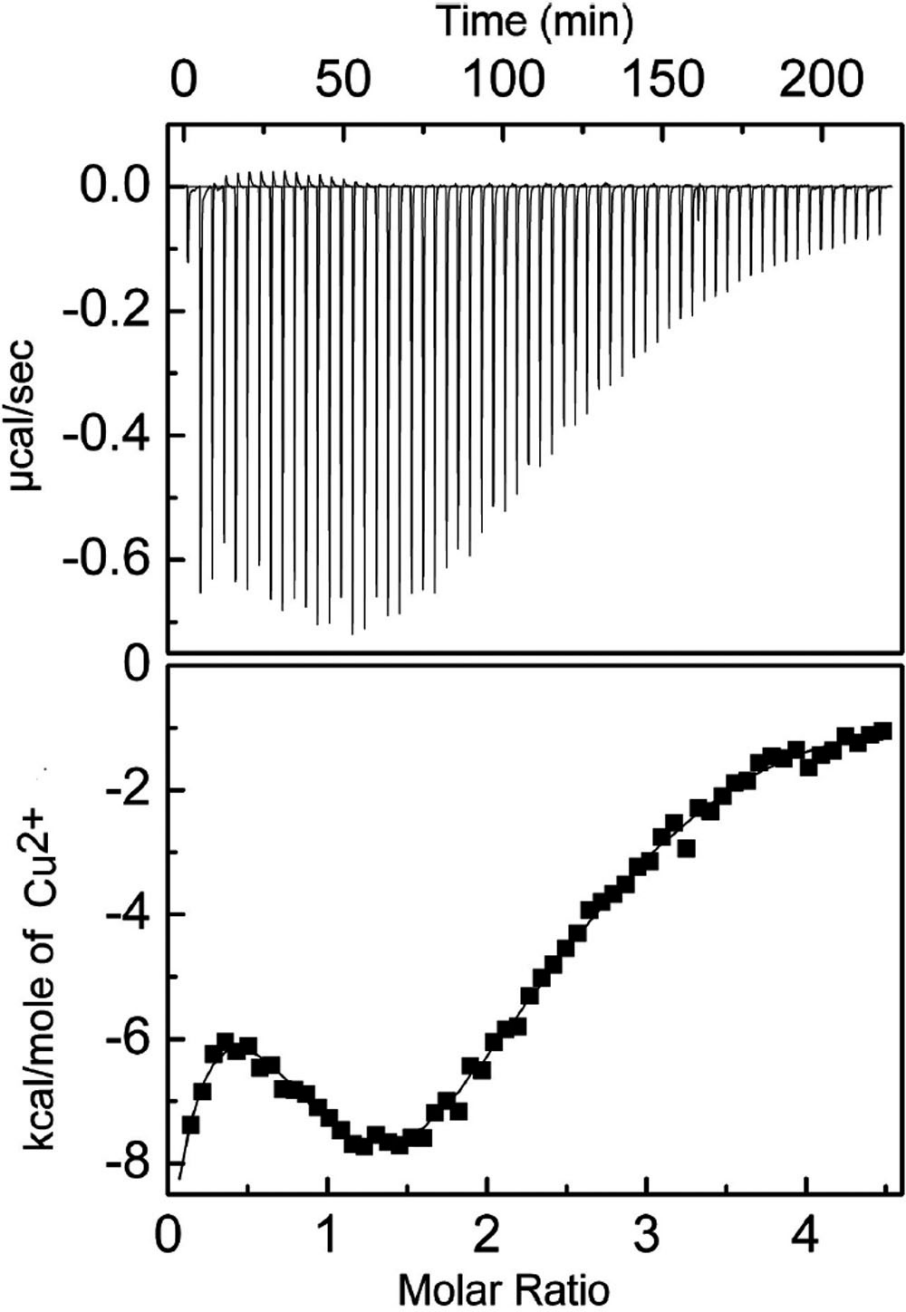
ITC measurements of Cu^2+^-binding to the mutant G98R αA-crystallin. The upper panel shows isotherms of enthalpic changes in mutant G98R αA-crystallin upon Cu^2+^ binding. The lower panel shows the fitted curve indicating molar heat values as a function of the Cu^2+^ to protein molar ratio. Measurements were made at 30 °C. The binding isotherm of G98R αA-crystallin exhibits the sequential mode of binding with five sets of binding sites: K1=4.98 (±0.3)×10^5^; ΔH1=-9740±326; ΔS1=-6.07; K2=3.22 (±0.2)×10^5^; ΔH2=8853±1480; ΔS2=54.4; K3=9.23 (±0.62)×10^4^; ΔH3=-1.0 (±0.06)×10^5^; ΔS3=-308; K4=7.58 (±0.6)×10^4^; ΔH4=2.012 (±0.13)×10^5^; ΔS4=686; K5=4.0 (±0.3)×10^5^; ΔH5=-1.337 (±0.09)×10^5^; ΔS5=-415.

The K_d(app)_ from fluorescence quenching studies was obtained as described in the Methods section. The overall dissociation constant, K_d(app)_, from ITC results was calculated from the association constants (see legends to Figure 2) using the formula, K_d_=[1/(K1*K2*K3*….Kn)^1/n^]. Since the observed K_d(app)_ is the net result of competition between Cu^2+^-protein interaction and Cu^2+^-glycine interactions, K_d(real)_ was estimated as described previously [37] as the product of K_d(app)_ and the first dissociation constant of Cu(Gly)_2_ (2.6×10^−6^ M).

An ITC experiment with G98R αA-crystallin resulted in large net exothermic heat changes exhibiting characteristic binding isotherms upon the addition of Cu^2+^ (Figure 2). The isotherm could be best fitted with sequential mode of binding with five sets of binding sites (parameters are given in the legend to Figure 2). Our earlier study has shown that αA-crystallin exhibits the sequential mode of binding to Cu^2+^ with three sets of binding sites [8]. The apparent differences in the number of sequential sets of sites between αA-crystallin and G98R αA-crystallin could be due to the differences in their Cu^2+^-induced structural changes, which contribute to the observed heat changes. However, the overall dissociation constants, K_d(app)_, obtained from ITC data and fluorescence quenching are comparable (Table 1). The real dissociation constants, K_d(real)_, obtained from K_d(app)_ (see Table 1) for αA-crystallin and G98R αA-crystallin reveal picomolar affinity for Cu^2+^. Thus, αA-crystallin and G98R αA-crystallin exhibit only marginal differences, if any, in their affinity to Cu^2+^.

### Redox-silencing of Cu^2+^ by αA- and G98R αA-crystallins

**Figure 3:**
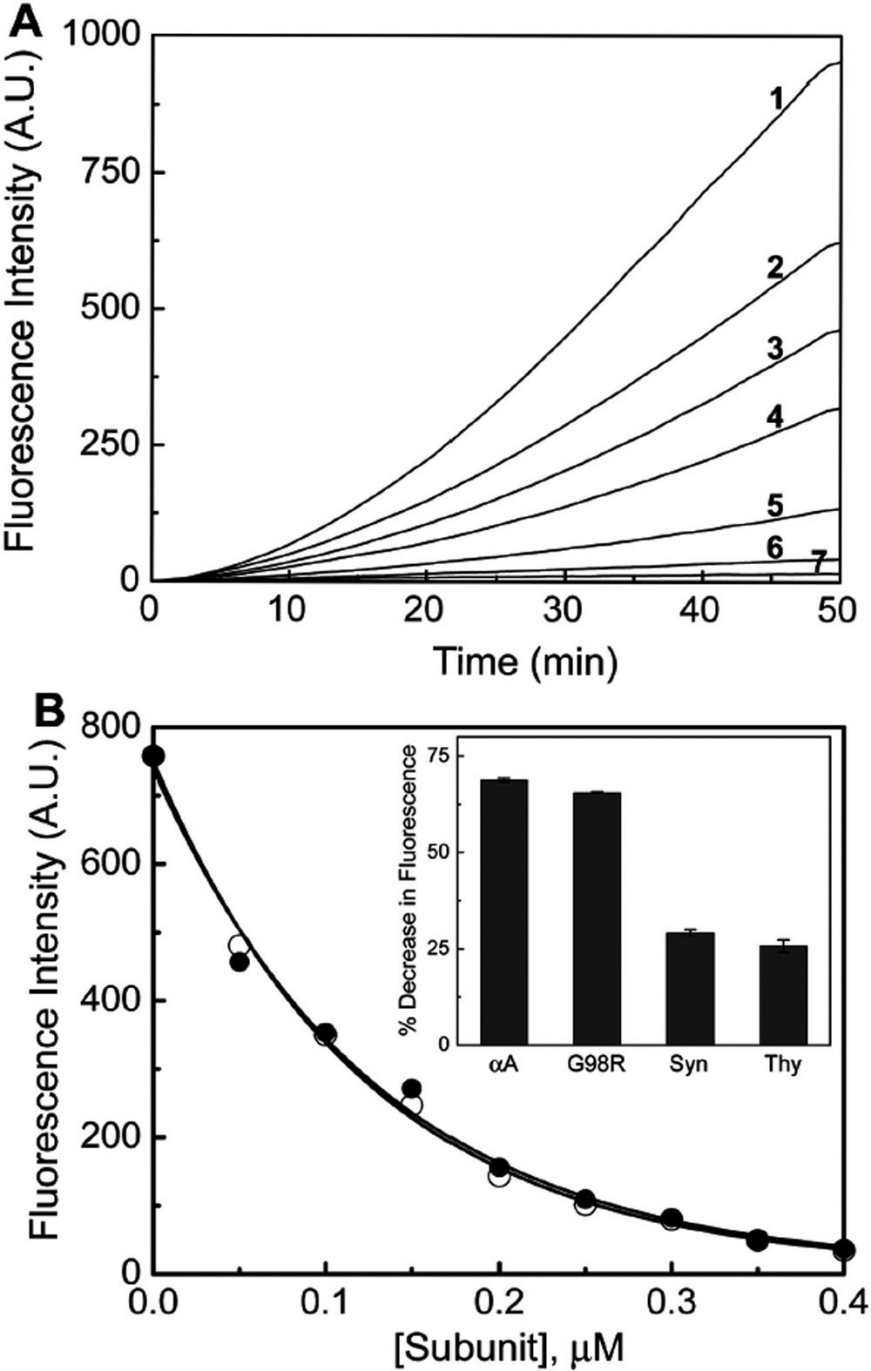
Redox-silencing of Cu^2+^ by αA-crystallin and G98R αA-crystallin. **A**: Cu^2+^-ascorbate-mediated OH**˙** generation in the absence (curve 1) and in the presence of 0.05, 0.1, 0.15, 0.25, and 0.4 μM αA-crystallin (curves 2–6). Curve 7 shows the trace of blank sample (in absence of protein and Cu^2+^). **B**: A decrease in coumarin fluorescence intensity (reflecting the inhibition of OH**˙** generation) is shown after 41.6 min as a function of concentration of αA- (○) and G98RαA-crystallin (●). Inset shows the percent decrease in fluorescence in the presence of 3 μg/ml α-crystallins, α-synuclein (Syn), and thyroglobulin (Thy). The results indicate that G98R mutation in αA-crystallin does not affect its redox-silencing property. Error bars for four experiments are also shown.

We have studied the effect of αA- and G98R αA-crystallins on the Cu^2+^-catalyzed, ascorbate-mediated generation of ROS. We have probed the generation of OH**˙** using coumarin-3-carboxylic acid (3-CCA), a non-fluorescent molecule that gets hydroxylated and becomes fluorescent [38]. Figure 3A shows that αA-crystallin inhibits the increase in fluorescence intensity effectively. Figure 3B shows that both αA- and G98R αA-crystallin inhibit the generation of hydroxyl radicals with comparable efficiencies. However, both thyroglobulin and α-synuclein, a Cu^2+^-binding protein, and thyroglobulin (which were used as controls), inhibited the generation of hydroxyl radicals to a very small extent (Figure 3B).

We have also monitored the generation of ROS using the fluorescent dye, fluorescein, whose fluorescence decreases upon oxidation by ROS [39]. Like wild type αA-crystallin, G98R αA-crystallin inhibits the generation of ROS significantly by inhibiting the Cu^2+^-induced oxidation of ascorbate itself (data not shown). Thus, αA-crystallin and G98R αA-crystallin exhibit a similar redox-silencing property.

### G98R αA-crystallin and the mixed oligomer exhibit increased propensity to Cu^2+^-induced self-aggregation

**Figure 4:**
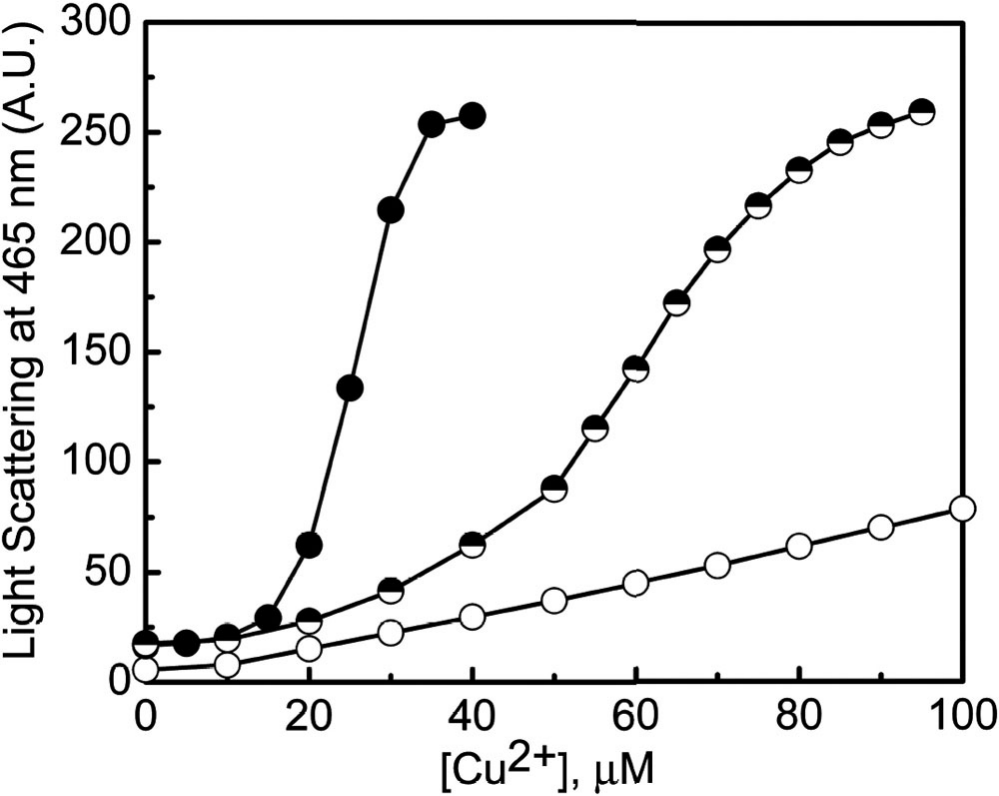
Cu^2+^-induced self-aggregation of αA-crystallin, G98R αA-crystallin, and their mixed oligomer. Aggregation of 0.1 mg/ml of αA-crystallin (○), G98R αA-crystallin (●), and their mixed oligomer (◓) as a function of increasing Cu^2+^ concentration at 37 °C in buffer B was monitored by light scattering at 465 nm expressed in arbitrary units (AU). G98R αA-crystallin and its mixed oligomer with wild type protein exhibit increased vulnerability to Cu^2+^-induced self-aggregation.

G98R αA-crystallin (in buffer A) becomes turbid above 50 μM Cu^2+^ whereas αA-crystallin starts aggregating only above 200 μM Cu^2+^. This tendency to aggregate is more pronounced in buffer B. Therefore, we have investigated the relative Cu^2+^-induced self-aggregation propensities of the wild type and mutant proteins in buffer B using light scattering at 465 nm (Figure 4). The light scattering of the αA-crystallin sample (0.1 mg/ml, approximately 5 μM subunits) increases gradually as a function of Cu^2+^ concentration (Figure 4). On the other hand, the light scattering of the G98R αA-crystallin sample increases sharply above 18 μM and saturates at around 40 μM, clearly demonstrating the higher propensity of G98R αA-crystallin to self-aggregate upon binding to Cu^2+^. We have also studied the self-aggregation propensity of the mixed oligomer (1:1 ratio of αA- and G98R αA-crystallin) with increasing concentrations of Cu^2+^ (Figure 4). The mixed oligomer exhibits a large increase in light scattering above 40 μM Cu^2+^. Thus, both G98R αA-crystallin and the mixed oligomer exhibit increased vulnerability to Cu^2+^-induced self-aggregation. However, mixed oligomer formation leads to a shift in the critical Cu^2+^ concentration (above which self-aggregation is pronounced) from 18 μM (G98R αA-crystallin alone) to about 50 μM.

### Reversibility of Cu^2+^-binding and induced aggregation of αA-, G98R αA-crystallins, and the mixed oligomer

We have investigated whether the observed Cu^2+^-induced changes in the fluorescence and aggregation properties are reversible. When we treated Cu^2+^-bound αA-crystallin, G98R αA-crystallin, and their mixed oligomers with 0.2 mM EDTA, about 89%, 73%, and 73%, respectively, of the observed fluorescence quenching was recovered (data not shown). This indicated that protein-bound Cu^2+^ could be dislodged by the metal ion chelators (albeit requiring more than the stoichiometric concentrations).

**Figure 5:**
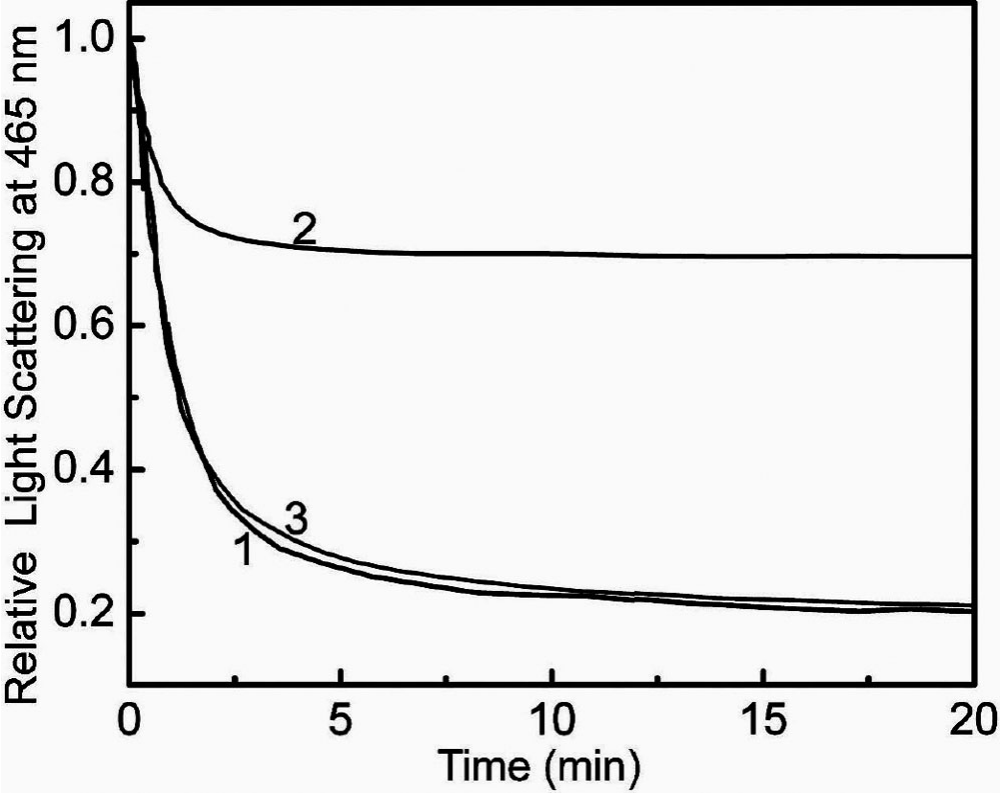
Reversibility of Cu^2+^-induced aggregation of αA-crystallin, G98R αA-crystallin, and their mixed oligomer. A solution containing 0.1 mg/ml of αA-crystallin, G98R αA-crystallin, or their mixed oligomer in buffer B was incubated for 30 min at 37 °C with 90, 30, or 90 μM of Cu^2+^, respectively. Reversibility of Cu^2+^-induced aggregation was monitored by relative decreases in light scattering of this solution after the addition of EDTA (200 μM). αA-crystallin is curve 1, G98R αA-crystallin curve 2, and their mixed oligomer curve 3. Cu^2+^-induced self-aggregation of G98RαA-crystallin is irreversible whereas that of αA-crystallin and the mixed oligomer is largely reversible.

We then investigated whether Cu^2+^-induced self-aggregation of these proteins exhibits reversibility. The small increase in light scattering observed upon treating the sample of αA-crystallin with high concentrations of Cu^2+^ (e.g., 90 μM) is reversed (>80%) upon adding 0.2 mM EDTA (Figure 5). On the other hand, the pronounced aggregation exhibited by G98R αA-crystallin (even at 30 μM Cu^2+^) is only partially reversible (about 30%) upon adding EDTA (Figure 5). Mixed oligomer exhibits pronounced self-aggregation upon treating with 90 μM Cu^2+^, which is significantly reversible upon adding EDTA (Figure 5). These results indicate that the Cu^2+^-induced aggregation of the mutant G98R αA-crystallin is largely irreversible.

### Cu^2+^-induced conformational changes in αA- and G98R αA-crystallins

To investigate conformational changes in αA- and G98R αA-crystallins upon binding to Cu^2+^, we performed circular dichroism and DLS experiments at the highest Cu^2+^ concentration at which Cu^2+^-induced aggregation is minimal.

**Figure 6:**
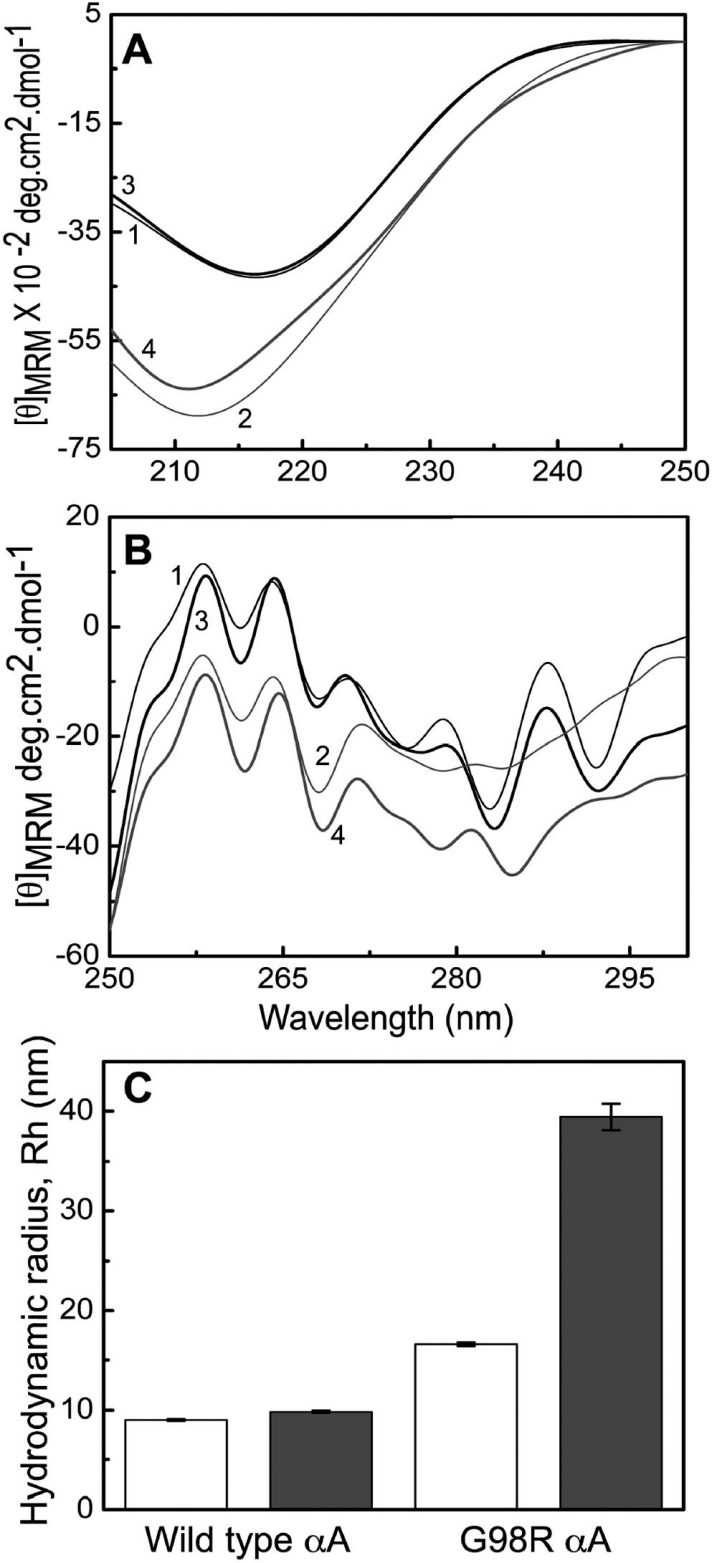
Cu^2+^-induced structural changes of αA- and G98R αA-crystallin. Far-UV (**A**) and near-UV (**B**) CD spectra of 1 mg/ml of αA-crystallin (curve 1) and G98R αA-crystallin (curve 2) and of 150 μM Cu^2+^-treated samples of αA-crystallin (curve 3) and G98R αA-crystallin (curve 4) in buffer B are shown. **C**: Changes in the mean hydrodynamic radii (R_h_) of 0.5 mg/ml αA-crystallin and G98R αA-crystallin in the absence (open bars) and in the presence of 75 μM of Cu^2+^ (filled bars) were determined by dynamic light scattering studies. The error bars represent the statistical variations of the mean hydrodynamic radii of αA-crystallin or mutant αA-crystallin between 10 experimental data. G98R αA-crystallin is more susceptible to the Cu^2+^-induced structural changes compared to αA-crystallin. [θ]_MRM_, mean residue mass ellipticity.

The far-UV CD spectrum of wild type αA-crystallin almost completely overlaps with that of the Cu^2+^-bound form, showing that the far-UV CD spectrum does not significantly change upon binding to Cu^2+^ under the experimental conditions (Figure 6A). G98R αA-crystallin exhibits increased ellipticity compared to αA-crystallin, indicating distinct structural differences. The far-UV CD spectrum of Cu^2+^-bound G98R αA-crystallin exhibits slightly decreased ellipticity compared to that of G98R αA-crystallin in the absence of Cu^2+^ (Figure 6A).

The near-UV CD spectra of αA-crystallin and the Cu^2+^-bound form of αA-crystallin (Figure 6B) show subtle differences in the 270–295 nm region (where tryptophan/tyrosine residues contribute to the chirality). G98R αA-crystallin shows significant differences in this region with loss of fine structure compared to αA-crystallin, indicating significant tertiary structural perturbations upon mutation. Moreover, the near-UV CD spectrum of the Cu^2+^-bound G98R αA-crystallin differs significantly from that of the protein in the absence of Cu^2+^, indicating that G98R αA-crystallin is more susceptible to Cu^2+^-induced tertiary structural changes compared to αA-crystallin.

DLS studies (Figure 6C) show that wild type αA-crystallin exhibits a mean hydrodynamic radius, R_h_, of ~9 nm, which increases to 9.8 nm when treated with Cu^2+^. G98R αA-crystallin exhibits higher R_h_ (16.5 nm) than wild type αA-crystallin, which increases dramatically to ~40 nm upon being treated with Cu^2+^ (Figure 6C). Thus, CD and DLS studies show that the structural changes (particularly in the tertiary and quaternary structure) induced by Cu^2+^ are more pronounced in G98R αA-crystallin than in αA-crystallin.

### Effect of Cu^2+^-binding on thermostability of αA- and G98R αA-crystallins

**Figure 7:**
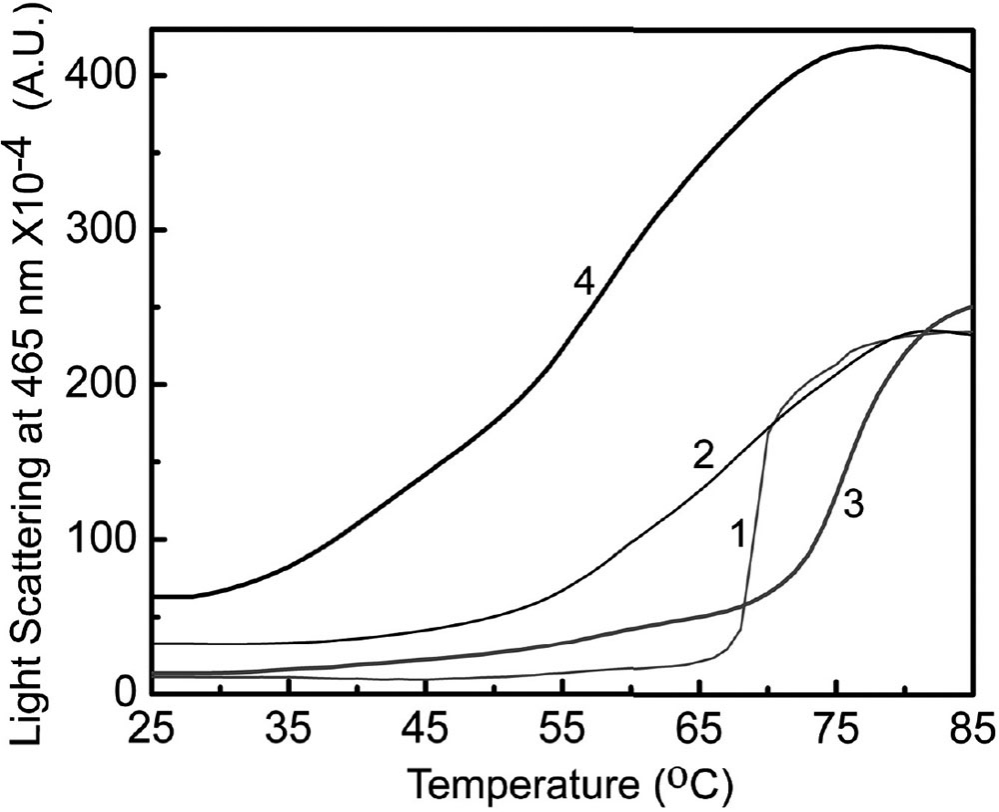
Thermal stability of wild type and G98R αA-crystallin upon Cu^2+^-binding. Aggregation of 0.2 mg/ml of αA-crystallin (curve 1) and G98R αA-crystallin (curve 2) in buffer B and of 30 μM Cu^2+^-treated samples of αA-crystallin (curve 3) and G98R αA-crystallin (curve 4) is shown. The aggregation was monitored by light scattering at 465 nm as a function of temperature. G98R mutation in αA-crystallin leads to decreased thermal stability upon Cu^2+^-binding.

α-crystallins in general are highly thermostable with respect to large unfolding of their secondary structural contents [5,40,41]. However, they show a transition around 60 °C exhibiting hydrophobicity changes [5,40-42]. Therefore, we have studied the thermostability by monitoring light scattering. αA-crystallin exhibits a sharp (cooperative) transition in light scattering around 66 °C (Figure 7). In the presence of Cu^2+^, the light scattering profile of αA-crystallin exhibits a gradual increase till about 68 °C and exhibits a sharp transition with an inflection point around 76 °C, indicating that Cu^2+^-binding stabilizes αA-crystallin against heat-induced self-aggregation. In conformity with our earlier observations [32,33], the light scattering of G98R αA-crystallin increases above 50 °C in a less cooperative manner (Figure 7). Interestingly, the light scattering profile of the Cu^2+^-bound G98R αA-crystallin increases around 35 °C, which is more pronounced above 55 °C. Thus, Cu^2+^-binding further destabilizes G98R αA-crystallin against heat-induced aggregation.

### Effect of Cu^2+^ on the chaperone-like activity

**Figure 8:**
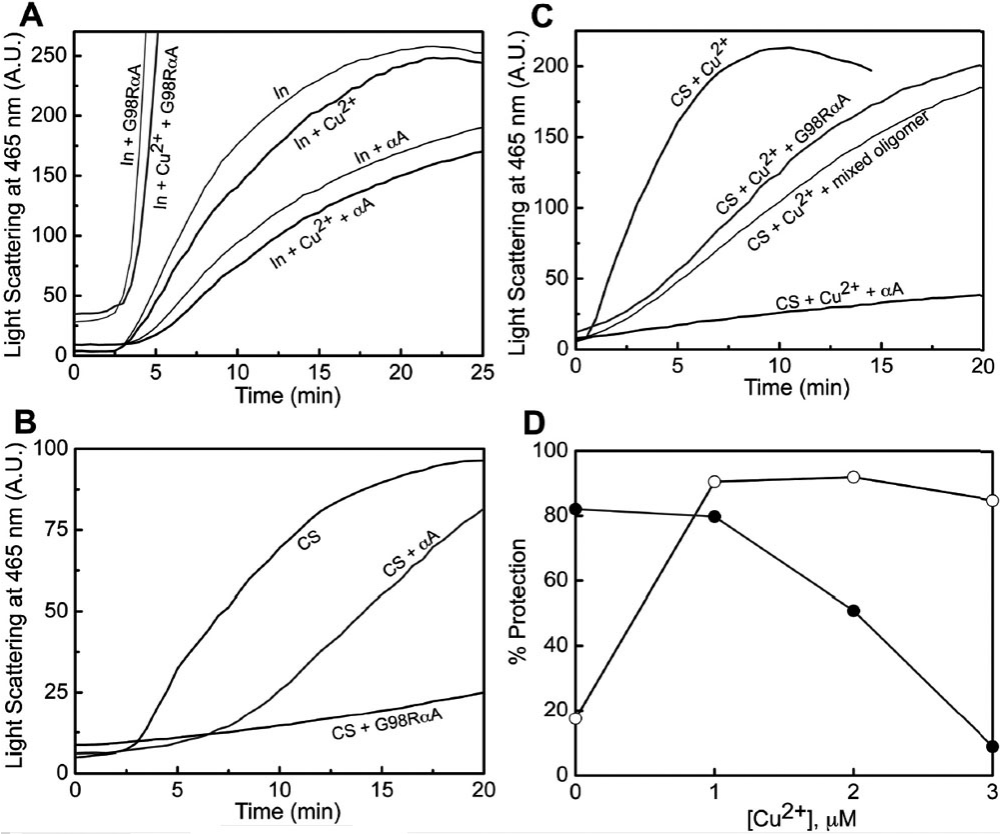
Chaperone-like activity of αA-crystallin and G98R αA-crystallin with and without Cu^2+^ using insulin and citrate synthase as target proteins. The difference in the chaperone-like activity of the mutant protein with respect to the wild type protein toward DTT-induced aggregation of insulin at 37 °C and heat-induced aggregation of CS at 43 °C was assayed in the absence and the presence of Cu^2+^. **A**: Aggregation of 0.2 mg/ml insulin (In) in 10 mM phosphate buffer (pH 7.4) containing 100 mM NaCl was monitored by light scattering at 465 nm (expressed in arbitrary units [AU]) in the absence or in the presence of 0.1 mg/ml αA-crystallin and G98R αA-crystallin. A similar experiment was performed in the presence of 15 μM Cu^2+^. **B**: Aggregation of 25 μg/ml citrate synthase (CS) was monitored by light scattering at 465 nm in the absence and in the presence of 20 μg/ml of either αA-crystallin or G98R αA-crystallin. **C**: The effect of αA-crystallin, G98R αA-crystallin, and their mixed oligomer on the aggregation of CS in the presence of 3 μM Cu^2+ ^was measured. **D**: Percentage protection of CS aggregation in the presence of 1 μM αA-crystallin (○) and G98RαA-crystallin (●) as a function of Cu^2+^ concentration indicate that the intrinsic chaperone ability of αA-crystallin is increased and that of G98R αA-crystallin is decreased. The experiments were performed three times, and the trends were reproducible. Representative data are shown.

We have earlier shown that G98R αA-crystallin does not prevent DTT-induced aggregation of insulin but co-aggregates with the target protein [32,33]. We have investigated the effect of Cu^2+^ (15 μM) on the chaperone-like activity of αA- and G98R αA-crystallin (0.1 mg/ml or ~5 μM subunit concentration) toward DTT-induced aggregation of insulin (Figure 8A) where Cu^2+^-induced aggregation is minimal. Ganadu et al. [9] have reported that Cu^2+^ increases the chaperone-like activity of αB-crystallin toward DTT-induced aggregation of insulin. We found a marginal Cu^2+^-induced increase in the chaperone-like activity of αA-crystallin whereas G98R αA-crystallin lacks chaperone-like activity and there is no significant change in the presence of Cu^2+^ (Figure 8A).

We found that G98R αA-crystallin prevents thermal aggregation of CS better (82%) than αA-crystallin (18%) at the same concentration (Figure 8B). This observation is in agreement with a recent report by Murugesan et al. [34], indicating the target protein-dependent chaperone-like activity for G98R αA-crystallin. Addition of Cu^2+^ promotes aggregation of CS, and αA-crystallins efficiently suppress the aggregation (compare Figure 8B,C). The observed suppression of aggregation by αA-crystallin would have two components: (i) preferential binding of Cu^2+^, which prevents its adverse effect on CS, and (ii) the effect of Cu^2+^-binding on the intrinsic chaperone ability of αA-crystallins. If the first mechanism alone is responsible, one would expect light scattering profiles in the presence of the αA-crystallins with or without Cu^2+^ to overlap. Interestingly, the percentage protection of αA-crystallin increases from 18% in the absence of Cu^2+^ to ~90% in the presence of Cu^2+^ (Figure 8B-D), clearly showing that Cu^2+^-binding significantly increases its intrinsic chaperone ability. On the other hand, Cu^2+^-binding drastically decreases the chaperone ability of G98R αA-crystallin from ~82% in the absence of Cu^2+^ to below 10% at 3 μM Cu^2+^ (Figure 8B-D). It may be noted that Cu^2+^-induced aggregation of G98RαA-crystallin is minimal at these concentrations of Cu^2+^.

Despite the enhanced chaperone activity of αA-crystallin in the presence of Cu^2+^, mixed oligomers having equimolar concentration of αA- and G98R αA-crystallins exhibit decreased chaperone property in the presence of Cu^2+^ as observed in the case of the mutant protein alone (Figure 8C). Thus, mixed oligomer formation with wild type subunits does not significantly improve the adverse effect of Cu^2+^-binding on the chaperone property of the mutant subunits. Our earlier study also showed that the structural and chaperone property toward DTT-induced aggregation of insulin of the mixed oligomers are dominated by the mutant protein [33]. Thus, our study demonstrates that while Cu^2+^-binding increases the intrinsic chaperone ability of αA-crystallin, it decreases the chaperone ability of G98R αA-crystallin (Figure 8D).

## Discussion

Metal ions such as Cu^2+^ and/or Zn^2+^ have been implicated in neurodegenerative disorders such as Alzheimer, Parkinson, and prion diseases [43,44]. Increased levels of Cu^2+^, Cd^2+^, Zn^2+^, and Ca^2+^ are also known to be present in cataractous lenses [11-15], indicating that they are potential environmental risk factors. Oxidative damage is an important cause of posttranslational modifications in age-related cataracts [45-47]. Cu^2+^ is known to catalyze production of reactive oxygen species (ROS) in the presence of ascorbate [48,49], which in turn can lead to oxidation of amino acid side chains, protein fragmentation, and protein–protein cross-links. The Cu^2+^-binding properties of αA- and αB-crystallins and their redox-silencing activity seem to be another defense mechanism provided by the chaperone molecule [8]. In the present study, we have addressed the effect of Cu^2+^ as a potential environmental risk factor on the self-aggregation propensities and chaperone property of the wild type and the G98R αA-crystallin.

Our study shows that the wild type and the mutant protein only differ marginally in their Cu^2+^-binding and redox silencing properties. However, the consequences of the interaction of Cu^2+^ with wild type and G98R αA-crystallin are drastically different. G98R αA-crystallin is more vulnerable to Cu^2+^-induced self-aggregation than the wild type protein. Our earlier studies show that the G98R mutation results in a folding-defective protein. The mutant protein has altered secondary, tertiary, and quaternary structure and is aggregation-prone [32,33]. However, the formation of mixed oligomer of G98R αA-crystallin with wild type subunits prevents aggregation of the mutant protein [33]. Our study shows that G98R αA-crystallin as well as its mixed oligomers with subunits of wild type αA-crystallin is more susceptible to Cu^2+^-induced structural changes and self-aggregation compared to αA-crystallin.

Besides Cu^2+^, elevated levels of Zn^2+^ and Cd^2+^ have been reported in cataractous lenses [11-15]. Cigarette smoking, a lifestyle habit, is considered a risk factor in cataractogenesis [50], and it is shown to significantly increase accumulation of lenticular Cd^2+^ as well as Cu^2+^ [15]. Biswas and Das [10] have reported that Zn^2+^ increased the chaperone-like activity of αA- and αB-crystallins toward β-mercaptoethanol-induced aggregation of insulin. We also made a similar observation that the chaperone-like activity of αA-crystallin toward the aggregation of CS is significantly increased in the presence of Zn^2+^ as well as Cd^2+^ (data not shown). On the other hand, Zn^2+^ and Cd^2+^, like Cu^2+^, decreased the chaperone activity of G98R αA-crystallin (data not shown). We have observed that that Zn^2+^ and Cd^2+^ also promoted the self-aggregation of G98R αA-crystallin and the mixed oligomers (data not shown). Columbic interactions (ionic interactions and coordination complex) of these metal ions could affect the wild type and mutant proteins differentially, thereby exhibiting differences in their structural properties, propensities to self-aggregate, stability, and chaperone activities. It is important to note that the effect of these metal ions (Cu^2+^, Cd^2+^, and Zn^2+^) on the properties of the mutant protein appears to be specific as Ca^2+^ (even at very high concentration of 5 mM) does not cause self-aggregation of αA- or G98R αA-crystallin or their mixed oligomers (data not shown). Further, Ca^2+^ (even at the metal ion to protein ratio of 500:1 [M/M]) does not significantly alter their chaperone-like activity toward the aggregation of CS (data not shown).

Thus, our study shows that G98R αA-crystallin is more vulnerable to heavy metal ions such as Cu^2+^, Cd^2+^, and Zn^2+^ than wild type αA-crystallin. At lower concentrations of Cu^2+^, Cd^2+^, and Zn^2+^ (where aggregation does not occur), the chaperone activity of G98R αA-crystallin is decreased drastically whereas that of wild type αA-crystallin increases significantly. Higher concentrations of these metal ions increase the propensity of the mutant protein to self-aggregate. It is possible that structural alteration of the mutant protein [32,33] and metal binding together increase its self-aggregation propensity. αA-crystallin exists predominantly in the cortical region of the lens [51]. A gradient of Cu^2+^ exists in the lens with the highest concentration being in the cortical region [12]. The G98R mutation leads to the ring-like opacity at the age of 16 years before becoming full blown cataract [22]. It is possible that these independent observations have some link. Our study may prove useful in understanding how factors such as metal ions could augment the phenotype in the genetically predisposed condition.
